# Suppression of adult cytogenesis in the rat brain leads to sex‐differentiated disruption of the HPA axis activity

**DOI:** 10.1111/cpr.13165

**Published:** 2021-12-30

**Authors:** Tiago Silveira‐Rosa, António Mateus‐Pinheiro, Joana Sofia Correia, Joana Margarida Silva, Joana Martins‐Macedo, Bruna Araújo, Ana Rita Machado‐Santos, Nuno Dinis Alves, Mariana Silva, Eduardo Loureiro‐Campos, Ioannis Sotiropoulos, João Miguel Bessa, Ana João Rodrigues, Nuno Sousa, Patrícia Patrício, Luísa Pinto

**Affiliations:** ^1^ Life and Health Sciences Research Institute (ICVS) School of Medicine University of Minho Braga Portugal; ^2^ ICVS/3B’s ‐ PT Government Associate Laboratory Braga/Guimarães Portugal; ^3^ Department of Internal Medicine Coimbra Hospital and University Center Coimbra Portugal; ^4^ Bn’ML – Behavioral and Molecular Lab Braga Portugal; ^5^ Present address: Department of Psychiatry Columbia University New York New York USA; ^6^ Present address: New York State Psychiatric Institute New York New York USA

**Keywords:** corticosterone, hippocampal cytogenesis, HPA axis, sex differences, stress

## Abstract

**Objectives:**

The action of stress hormones, mainly glucocorticoids, starts and coordinates the systemic response to stressful events. The HPA axis activity is predicated on information processing and modulation by upstream centres, such as the hippocampus where adult‐born neurons (hABN) have been reported to be an important component in the processing and integration of new information. Still, it remains unclear whether and how hABN regulates HPA axis activity and CORT production, particularly when considering sex differences.

**Materials and Methods:**

Using both sexes of a transgenic rat model of cytogenesis ablation (GFAP‐Tk rat model), we examined the endocrinological and behavioural effects of disrupting the generation of new astrocytes and neurons within the hippocampal dentate gyrus (DG).

**Results:**

Our results show that GFAP‐Tk male rats present a heightened acute stress response. In contrast, GFAP‐Tk female rats have increased corticosterone secretion at nadir, a heightened, yet delayed, response to an acute stress stimulus, accompanied by neuronal hypertrophy in the basal lateral amygdala and increased expression of the glucocorticoid receptors in the ventral DG.

**Conclusions:**

Our results reveal that hABN regulation of the HPA axis response is sex‐differentiated.

## Graphical abstract

Cytogenesis ablation in the adult brain of female rats leads to HPA axis disruption. Female, but not male, GFAP‐Tk rats show increased corticosterone secretion at nadir, a heightened, yet delayed, response to an acute stress, neuronal hypertrophy in the basal lateral amygdala and increased glucocorticoid receptor expression in the ventral DG. These results reveal a sex‐differentiated hABN regulation of the HPA axis response.

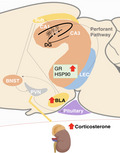

## 1 INTRODUCTION

Stress response and stress hormones are a crucial part of an intrinsic and beneficial physiological mechanism. It is through the action of stress hormones, mainly glucocorticoids (cortisol in humans; corticosterone (CORT) in rodents), that the systemic response to stressful events is started and coordinated. Besides this phasic response to environmental changes, glucocorticoids also have a basal, tonic function within the circadian rhythm.^1^ Circadian fluctuation of all circulating adrenal hormones, including CORT, is coordinated by the ‘pacemaker’ activity of the suprachiasmatic nucleus of the hypothalamus. This regulation occurs through direct modulation of the hypothalamic‐pituitary‐adrenal (HPA) axis activity and through the paraventricular nucleus signalling through the autonomic nervous system splanchnic nerve to the adrenal gland, which in turn also regulates adrenocorticotrophic hormone (ACTH) sensitivity.

In response to environmental changes, HPA axis activity is predicated on information processing by upstream centres, such as the medial prefrontal cortex (mPFC) and hippocampus, whose activity modulates the HPA axis, both in basal and in stressful conditions.^2^

The hippocampus is one of the few known brain regions where new cells, neurons and astrocytes are actively generated from resident neural stem cells (NSCs) in adulthood, under physiological conditions. Within the hippocampus, their adult cytogenesis process occurs in the subgranular zone of the dentate gyrus (DG). Hippocampal adult‐born neurons (hABN) have unique properties while undergoing their maturation process, such as increased synaptic plasticity and enhanced electrophysiological properties.^3^, ^4^, ^5^, ^6^ Curiously, recent data have also shown that even after a 6‐week period of maturation, hABN present morphological features that distinguish them from the neonatal‐born neuronal population.^7^ Functionally, hABN are an important component in the processing and integration of new information, being involved in cognitive behaviours, particularly pattern separation^8^ and in the stress response, where they play a role in regulating endocrinology and behaviour‐related stress reactivity.^9^, ^10^ When homeostasis is not achieved after stress response, maladaptive phenotypes may emerge. Studies looking into dysfunctional stress responses have identified at least a triad of effects: reduced hippocampal neurogenesis, HPA axis hyperactivity and high CORT levels.^11^, ^12^, ^13^ Because these disruptions are, to some extent, interdependent, they may create a feedforward loop that exacerbates future responsiveness to environmental challenges, contributing to the emergence of psychiatric disorders (eg generalized anxiety disorder and major depressive disorder [MDD]). In animal models, this disruptive loop is typically achieved either through chronic stress protocols or through exogenous delivery of CORT.^13^, ^14^, ^15^ However, it is still unclear whether and how hippocampal cytogenesis regulates the HPA axis activity and CORT production, especially when considering sex differences.^16^ For instance, female rodents have greater expression of CORT and HPA axis in response to novel stress stimuli,^17^ highlighting the importance of including both sexes when analysing the HPA axis and its modulators. Herein, we used both males and females of a transgenic rat model of cytogenesis suppression to assess whether and how reducing cytogenesis in the adult mammalian brain impacts HPA axis function and CORT production, as well as its repercussions for neuromorphology and behaviour.

## 2 MATERIALS AND METHODS

### 2.1 Animals

A transgenic rat line expressing the HSV‐thymidine kinase (Tk) under the human GFAP promoter (GFAP‐Tk) and generated on a Sprague Dawley background was kindly provided by Dr. Jonathan Flint.^18^ Animals were bred at the Life and Health Sciences Research Institute (ICVS) animal facility from 2 female GFAP‐Tk founders using Sprague Dawley WT males (Charles River, Barcelona, Spain). Female GFAP‐Tk rats were used for breeding as males are infertile. Rats were weaned at 21–28 days of age, group‐housed in polypropylene cages (2 per cage), genotyped by PCR and kept under standard laboratory conditions (12‐h/12‐h light‐dark cycles, lights on at 8 am, 22°C, relative humidity of 55%, food and water *ad libitum*). A 20% loss on total body mass was defined as the humane endpoint for animal experimentation, along with any other evident signs of significant animal's distress or compromised well‐being.

This work was conducted in accordance with the EU Directive 2010/63/EU and the Portuguese Veterinary Authority, Direção Geral de Alimentação e Veterinária (DGAV), recommendations on animal care and experimentation, following approval from the Ethics Subcommittee for the Life and Health Sciences (SECVS 068/2017), from the University of Minho.

### 2.2 Ganciclovir (GCV) treatment

Twelve‐ to fourteen‐week‐old GFAP‐Tk+/− (heterozygous for the mutation of interest) and WT littermates, males and females, were injected intraperitoneally (i.p.) with GCV (20 mg.kg^−1^; CAS 82410–32–0; Kemprotec Limited; in hydroxyethyl cellulose 0.5% w/v in ultra‐pure water) once a day, for 18 consecutive days, to promote cytogenesis ablation. Animals' weight and coat state score were monitored weekly as detailed in the Appendix S1.

### 2.3 Monitoring of the oestrous cycle

Oestrous cycle was monitored through vaginal cytology, as previously described.^19^ For that, vaginal smears were collected using inoculating loops in the weeks prior and after GCV injections. Vaginal smears were also performed immediately after anxiety and anhedonia behavioural tests. Females were evaluated for a week, to include at least one full oestrous cycle (4–5 days), and considered to have regular cycles if cycle changes were in accordance with previous reports of time estimates^20^ with a maximum deviation of 6h.

### 2.4 Corticosterone level quantification

**FIGURE 1:**
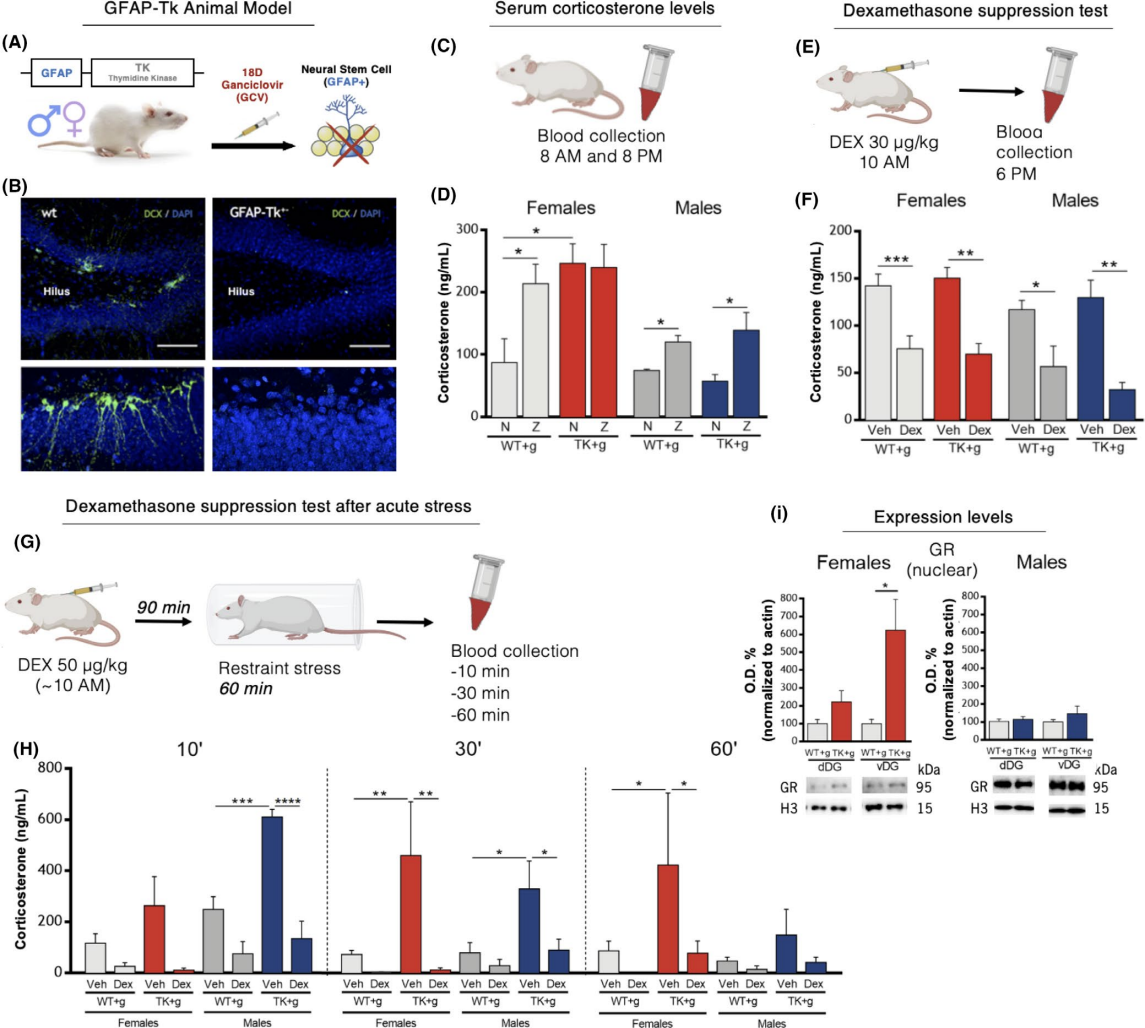
*Corticosterone and HPA axis assessment in a cytogenesis ablation model*. (A) Schematic depiction of the GFAP‐Tk rat model setup. (B) Ganciclovir (GCV) treatment eliminated all doublecortin (DCX) cells in the hippocampal DG. (C) Depiction of serum corticosterone (CORT) collection protocol. (D) CORT levels at nadir (N) and zenith (Z). GFAP‐Tk females show increased levels of CORT at N. (E) Depiction of dexamethasone (Dex) suppression test (DST). (F) CORT levels before and after suppression. (G) Depiction of DST after acute stress. (H) CORT levels in females and males at 10, 30 and 60 min after restraint stress. (I) Nuclear glucocorticoid receptor (GR) expression levels in the dorsal and ventral poles of the DG (dDG and vDG). Error bars denote s.e.m. *p < 0.05, **p < 0.01 and ***p < 0.001

Corticosterone levels were measured in the blood serum, using a commercially available ELISA kit (Abcam). Samples were run in duplicate. Blood was collected by tail venipuncture. Three independent experiments were performed 3 days after GCV treatment to assess the HPA axis function, as depicted in Figure 1C,E,G, and detailed in the Appendix S1, with adaptations from previous works.^9^, ^21^, ^22^

### 2.5 Behavioural analysis

Behavioural testing started 1 day after the end of GCV injections, following the sequence depicted in Figure 3A.

Anhedonia (SCT) was performed between 8 pm and 8 am (dark period), whereas anxiety‐like (novelty‐suppressed feeding [NSF], elevated plus maze [EPM], open field [OF]) and depressive‐like (forced swimming test, FST) behaviours were assessed during the period between 8 am and 8 pm (light period).

#### 2.5.1 Sucrose consumption test (SCT)

To assess anhedonia, baseline sucrose preference values were established during a 1‐week habituation period (1 week before the beginning of GCV treatment). Animals were presented with two pre‐weighed drinking bottles, one containing water and another with 2% (w/v) sucrose, as previously described.^23^ At the end of the GCV treatment, a single SCT trial was performed. Before testing, rats were food‐ and water‐deprived for 12 h and exposed to the drinking bottles for 1h (starting at the beginning of the dark period). Sucrose preference was calculated as previously described.^24^

#### 2.5.2 Novelty suppressed feeding (NSF) test

Anxiety‐like behaviour was assessed through the NSF test, as previously described.^25^ The latency to feed in the arena was used as a measure of anxiety‐like behaviour; the food consumption in the cages provided a measure of appetite drive.

#### 2.5.3 Elevated plus maze (EPM) and open field (OF)

The EPM and the OF were additionally used to assess anxiety‐like behaviour, as previously described.^26^

#### 2.5.4 Forced swimming test (FST)

Depressive‐like behaviour was assessed in the FST, as previously described.^25^ Depressive‐like behaviour was considered as an increase in the immobility time.

### 2.6 Immunohistochemical analysis

Animals (*n* = 5 per group) were deeply anaesthetized with sodium pentobarbital (20%; Eutasil; CEVA) and transcardially perfused with 0.9% saline solution followed by cold 4% paraformaldehyde (PFA). Brains were removed and post‐fixed in 4% PFA. Twenty‐four hours later, brains were transferred to phosphate‐buffered saline solution (PBS), for vibratome cutting.

Coronal vibratome sections (50 μm) were stained for doublecortin (DCX; goat polyclonal; 1:50; sc‐8066; Santa Cruz Biotechnology), for GCV‐induced cytogenesis ablation assessment. Sections were incubated with the appropriate secondary antibody (1:1000; donkey anti‐goat Alexa Fluor^®^ 488; A11055; Life Technologies, Thermo Fisher Scientific). All sections were stained with 4’,6‐diamidino‐2‐phenylindole (DAPI; 1 mg.ml^−1^; Invitrogen) and mounted with PermaFluor mounting medium (Thermo Scientific).

Imaging was performed by fluorescence microscopy (Olympus BX‐61; Olympus) or by confocal microscopy (Olympus FluoViewTM FV1000).

### 2.7 Western blotting‐subcellular fractionation

Animals (*n* = 5–7 per group) were deeply anaesthetized with sodium pentobarbital (20%; Eutasil; CEVA, France) and transcardially perfused with cold 0.9% NaCl. Brains were rapidly collected, and brain regions were macrodissected and immediately stored at –80°C. Biochemical fractionation and immunoblotting of dorsal and ventral hippocampus were analysed, as described in the Appendix S1.

### 2.8 Neuromorphology analysis

Animals (*n* = 4–5 per group) were deeply anaesthetized with sodium pentobarbital (20%; Eutasil; CEVA) and transcardially perfused with 0.9% saline solution. Brains were removed and post‐fixed in Golgi‐Cox solution for 2 weeks and then transferred to 30% sucrose solution for further processing, as previously described.^27^ Dendritic length and neuronal branching from dorsal and ventral hippocampal DG and basolateral amygdala (BLA) were assessed. For each animal, 7–10 neurons fulfilling previously described criteria were analysed per subregion.^28^ Dendritic branches were reconstructed at ×1,000 magnification on a motorized microscope (Axioplan 2; Carl Zeiss; for BLA samples and Olympus BX‐53; Olympus; for the remaining samples) and Neurolucida software (MBF Bioscience). Three‐dimensional analysis of the reconstructed neurons was performed using the NeuroExplorer software (MBF Bioscience). The number of dendritic branching was evaluated in 3D Sholl analysis, measuring the number of concentric radial intersections at intervals of 20 µm. Measures from individual neurons were averaged per animal and compared among experimental groups.

### 2.9 Statistical analysis

Statistical analysis was performed using Prism 6 (GraphPad Software, Inc.) and SPSS Statistics 26 (IBM). The underlying assumptions of all statistical procedures were assessed. Data normal distribution was tested using the Kolmogorov–Smirnov test. Homogeneity of variances was assessed with Levene's test when different groups were compared and with Mauchly's test of sphericity when repeated measures were compared. The extreme studentized deviate (ESD) method was used to determine significant outliers. Student's *t* test was used to assess differences between WT and GFAP‐Tk groups. Two‐way ANOVA was used to compare between different stages of the oestrous cycle in WT and GFAP‐Tk groups and to compare between sexes and treatments. Repeated‐measures ANOVA was used to assess differences in the body weight changes, in the Sholl analysis and in the DST after acute stress analyses. Test statistics and p‐values are shown for each test. Significance was set at *p* < 0.05. R‐squared for *t* test (R^sq^), and eta‐squared for ANOVA (Et^2^), is presented whenever statistical significance is reached. Statistical results are summarized in Table S2.

## 3 RESULTS

### 3.1 GFAP‐Tk female rats have disrupted basal corticosterone levels

The GFAP‐Tk male rat model has been previously shown to present decreased numbers of DCX^+^ cells in the hippocampal DG after GCV treatment.^18^, ^29^, ^30^ Here, we performed a visual inspection of the expression of the neuroblast marker DCX, validating that GFAP‐Tk female rats also display a notable decreased number of DCX^+^ cells in the DG (Figure 1A,B).

To analyse GFAP‐Tk rat's well‐being, we evaluated weight gain and coat state during and after GCV treatment, with no differences observed among groups (Figure S1A,B). The oestrous cycle of GFAP‐Tk females prior or after GCV treatment was kept regular and identical to WT females (Figure S1C).

Corticosterone (CORT) levels were evaluated in two time points: morning (8 am, Nadir, basal) and evening (8 pm, Zenith, peak) (Figure 1C,D). Female rats displayed higher CORT baseline and peak levels (Figure 1D; 2‐way ANOVA, for CORT at nadir: F(1,13) = 12.308, *p* = 0.004, Et^2^ = 0.49; for CORT at zenith: F(1,13) = 7.458, *p* = 0.017, Et^2^ = 0.37). WT and GFAP‐Tk male rats presented higher CORT levels at zenith comparing to nadir, as expected (Table S2). Female GFAP‐Tk rats showed increased levels of CORT at nadir compared with WT in the same time point (Figure 1D; unpaired two‐tailed *t* test, t = 3.264, df = 9, *p* = 0.010, R^sq^ = 0.54). Additionally, no differences were observed in CORT levels of GFAP‐Tk females, between nadir and zenith (Figure 1D; unpaired two‐tailed *t* test, t = 0.133, df = 11, *p* = 0.896), while WT females presented higher levels of CORT at zenith (Figure 1D; unpaired two‐tailed *t* test, t = 2.464, df = 7, *p* = 0.043, R^sq^ = 0.46). CORT levels in GFAP‐Tk females were also analysed at nadir taking into consideration their oestrous cycle phase. Results showed that genotype, but not oestrous cycle phase, was a differentiating factor among females at nadir (Figure S2A; 2‐way ANOVA, for genotype: F(1,5) = 12.805, *p* = 0.016, Et^2^ = 0.72; for cycle phase: F(1,5) = 0.031, *p* = 0.867). Altogether, the above data suggest that cytogenesis ablation produces a disruption in the HPA axis and higher CORT levels at nadir in females.

### 3.2 GFAP‐Tk rats show a sex‐differentiated response profile to acute stress

To dissect a possible underlying cause for the increased levels of CORT at nadir in GFAP‐Tk female rats, we performed DST under basal conditions and after acute stress exposure. Under basal conditions, DEX was able to suppress circulating corticosterone levels in both male and female groups, independently of cytogenesis ablation (Figure 1E,F; 3‐way ANOVA, for genotype: F(1,37) = 0.040, *p* = 0.842; for treatment: F(1,37) = 43.087, *p* < 0.001, Et^2^ = 0.54; for sex: F(1,37) = 4.826, *p* = 0.034, Et^2^ = 0.12).

Next, we performed DST after exposure to an acute restraint stressor and collected blood at 10, 30 and 60 min post‐stress (Figure 1G–I). Repeated‐measures analysis showed that genotype and treatment (but not sex) were differentiating factors (repeated‐measures ANOVA, for genotype: F(1,23) = 15.198, *p* = 0.001, Et^2^ = 0.40; for treatment: F(1,23) = 27.784, *p* < 0.001, Et^2^ = 0.55; and for sex: F(1,23) = 0.257, *p* = 0.617). Regardless of the sex, analysing only vehicle‐treated rats shows that GFAP‐Tk rats of both sexes display higher CORT levels when compared to their vehicle‐treated WT counterparts at all different periods of measurement (Figure 1H; repeated‐measures ANOVA, for genotype: F(1,10) = 10.932, *p* = 0.008, Et^2^ = 0.522; and for sex: F(1,10) = 0.007, *p* = 0.936). Because sex appears not to be a differentiating factor, we divided the subsequent analysis among sexes. In females, vehicle‐treated GFAP‐Tk rats showed higher CORT levels comparatively to vehicle‐treated WT and to dexamethasone‐treated rats (Figure 1H; repeated‐measures ANOVA, for genotype: F(1,15) = 9.003, *p* = 0.009, Et^2^ = 0.375; for treatment: F(1,15) = 18.695, *p* = 0.001, Et^2^ = 0.56; and for genotype*treatment: F(1,15) = 7.628, *p* = 0.015, Et^2^ = 0.34). When analysing each time point separately, CORT levels of vehicle‐treated GFAP‐Tk females are not different at the 10‐min time point from other female groups; however, their CORT levels are higher at the 30‐min time point and remain higher at 60‐min time point, when compared to all other female groups (Figure 1H; 60 min TP: 2‐way ANOVA, for genotype: F(1,17) = 5.838, *p* = 0.027, Et^2^ = 0.26; for treatment: F(1,17) = 6.399, *p* = 0.022, Et^2^ = 0.27; and for genotype*treatment: F(1,17) = 2.313, *p* = 0.147).

Vehicle‐treated GFAP‐Tk males have a similar general profile to females, displaying higher CORT levels when compared to vehicle‐treated WT and DEX‐treated rats (Figure 1I; repeated‐measures ANOVA, for genotype: F(1,8) = 8.387, *p* = 0.020, Et^2^ = 0.51; for treatment: F(1,8) = 13.145, *p* = 0.007, Et^2^ = 0.62; and for genotype*treatment: F(1,8) = 3.603, *p* = 0.094). Contrarily to female groups, vehicle‐treated GFAP‐Tk males presented higher CORT levels at the 10‐ and 30‐min time points when compared to all other male groups, which was no longer noticeable at the 60‐min time point (Figure 1I; 60 min TP: 2‐way ANOVA, for genotype: F(1,8) = 1.517, *p* = 0.253; for treatment: F(1,8) = 1.748, *p* = 0.223; and for genotype*treatment: F(1,8) = 0.499, *p* = 0.500).

Furthermore, we assessed the protein levels of the GR and HSP90, involved in the cytoplasmic stabilization and nuclear translocation of GR,^31^, ^32^ in the hippocampal DG. GFAP‐Tk females, but not males, presented increased levels of GR specifically in the ventral DG (vDG), when compared to WT rats (Figure 1J; unpaired two‐tailed *t* test, t = 2.578, df = 12, *p* = 0.024, R^sq^ = 0.36). HSP90 levels were also increased in GFAP‐Tk females in the vDG, but not in the dDG, in comparison with WT females (Figure S2B; unpaired two‐tailed *t* test, t = 2.1.98, df = 12, *p* = 0.048, R^sq^ = 0.29).

### 3.3 Cytogenesis ablation does not impact behaviour in females

**FIGURE 2:**
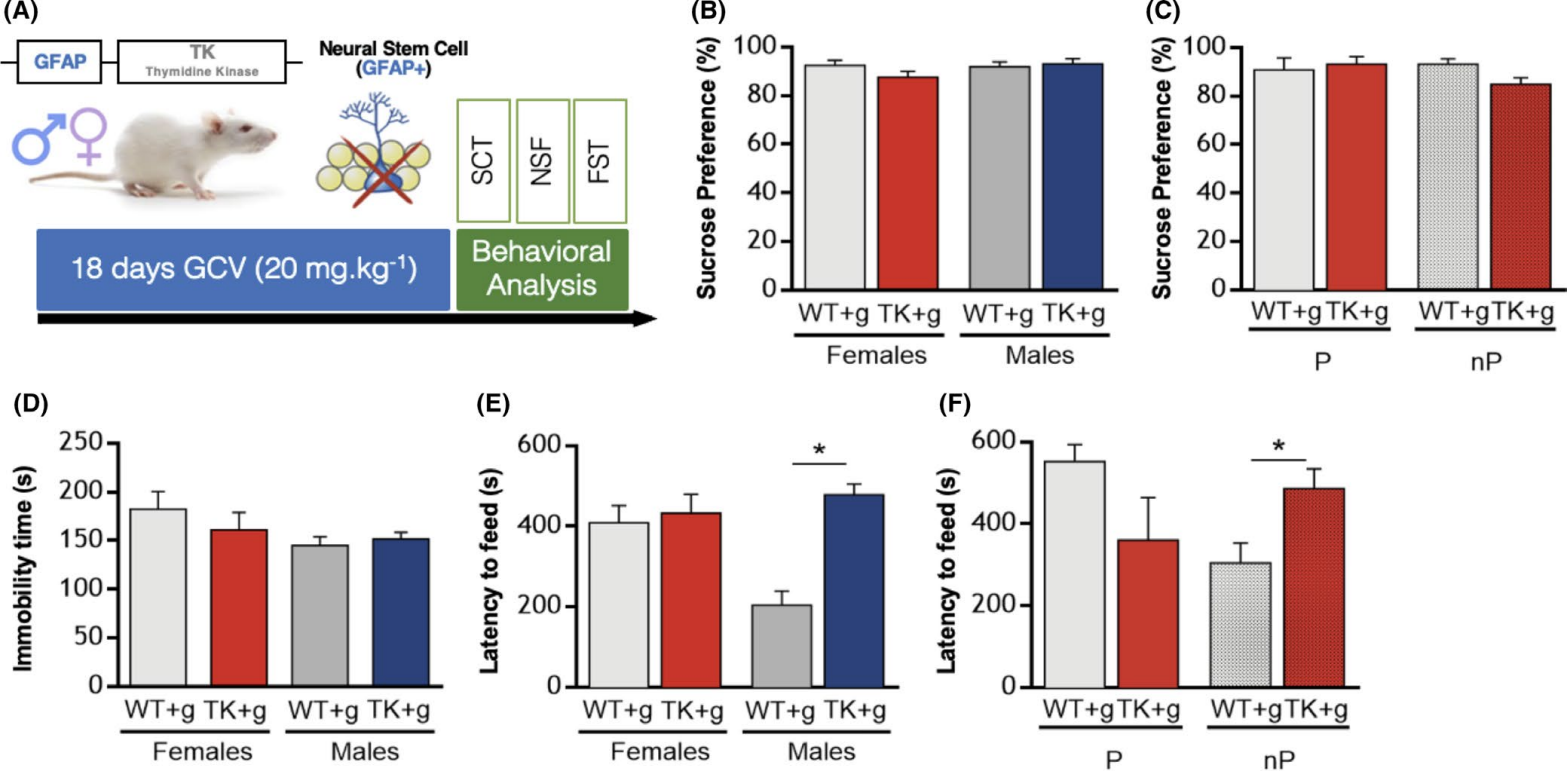
*Characterization of behavioural effects of adult cytogenesis ablation*. (A) Depiction of the experimental timeline, with behavioural assessment being performed immediately after cytogenesis ablation. Vaginal smears were performed immediately after behavioural tests, and females were divided into pro‐oestrous (P) and non‐pro‐oestrous (nP) groups. (B–C) Anhedonic behaviour was measured in the sucrose consumption test (SCT). (D) Depressive‐like behaviour, through learned helplessness, was measured in the forced swim test (FST). (E–F) Anxiety‐like behaviour was measured in the novelty‐suppressed feeding (NSF) test. Error bars denote s.e.m. *p < 0.05

Because cytogenesis ablation leads to increased levels of CORT specifically in female rats, we sought to evaluate whether this manipulation would also render a differential impact on behaviour (Figure 2A). Results are presented according to the phase in which the females were at the time of each test, either pro‐oestrous (P) or non‐pro‐oestrous (nP) phases. This division is based on previous reports that the elevation of progesterone and oestrogen levels during pro‐oestrus is capable of inducing either an anxiolytic^33^, ^34^, ^35^ or an anxiogenic effect.^36^, ^37^, ^38^, ^39^

Anhedonic behaviour, assessed in the SCT (Figure 2B,C), and depressive‐like behaviour, assessed in the FST (Figure 2D), were not altered in cytogenesis‐ablated female rats, in line with previous published descriptions in males^9^ and concordant with the male behavioural data from our laboratory,^30^ which is also presented here to allow for a side‐by‐side comparison of the impact of sex in behaviour.

In the NSF test, used to measure anxiety‐like behaviour, GFAP‐Tk males presented an increased latency to feed when compared to WT males (Figure 2E; unpaired two‐tailed *t* test, t = 6.2020, df = 17, *p* < 0.0001, R^sq^ = 0.69). Food consumption was unaffected, as previously described.^30^ Interestingly, GFAP‐Tk females presented an increased latency to feed only in non‐pro‐oestrous phases (2‐way ANOVA, for genotype: F(1,33) = 0.007, *p* = 0.933; for cycle phase: F(1,33) = 0.007, *p* = 0.933; and for genotype*cycle phase: F(1,33) = 10.116, *p* = 0.003, Et^2^ = 0.24), when compared to cycle‐matched WT females (Figure 2E,F; unpaired two‐tailed *t* test, t = 2.651 df = 22, *p* = 0.015, R^sq^ = 0.24). The food intake analysis revealed that GFAP‐Tk females' increased latency to feed in non‐pro‐oestrus was accompanied by a reduction in food consumption (Figure S3B; unpaired two‐tailed *t* test, t = 2.609, df = 22, *p* = 0.016, R^sq^ = 0.24). To further explore anxiety‐like behaviour in females, we performed two additional tests, the EPM and OF, in a new cohort of female rats. Due to the smaller size of this cohort (*n* = 14), it was not possible to explore the impact of the oestrous cycle on these behaviours. Both tests show no differences between genotypes (Figure S3C–E, Table S2), supporting that cytogenesis ablation does not impact anxiety‐like behaviour in female rats.

### 3.4 Cytogenesis ablation in female rats leads to hypertrophy of basolateral amygdala apical dendrites

**FIGURE 3:**
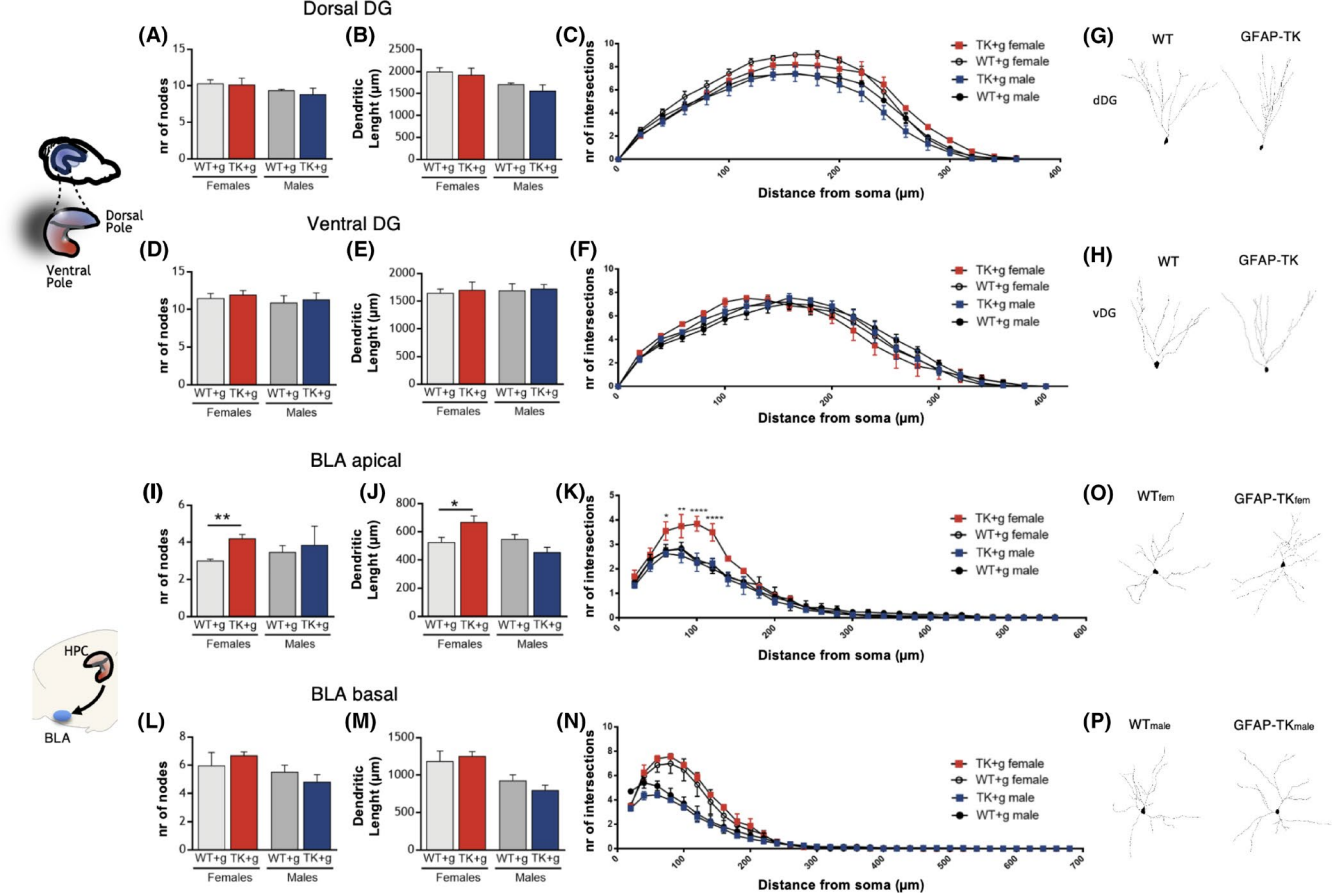
*Morphological assessment of dentate gyrus and basolateral amygdala neurons*. (A–C) Number of nodes (A), total dendritic length (B) and number of intersections (C) of dorsal dentate gyrus (dDG) granule neurons. (D) Representative neurons of dDG. (E–G) Number of nodes (E), total dendritic length (F) and number of intersections (G) of ventral dentate gyrus (vDG) granule neurons. (H) Representative neurons of vDG. (I–P) Basolateral amygdala (BLA) pyramidal neurons analysis was divided into apical dendrites (I–K) and basal dendrites (M–O). (I–K) Number of nodes (I), dendritic length (J) and number of intersections (K) of apical dendrites of BLA pyramidal neurons. (M–O) Number of nodes (M), dendritic length (N) and number of intersections (O) of basal dendrites of BLA pyramidal neurons. (L–P) Representative neurons of BLA. Error bars denote s.e.m. *p < 0.05

Given the disparate impact of cytogenesis ablation in CORT levels in females vs. males, we sought to evaluate the morphology of neurons in two brain regions involved in the regulation of the HPA axis, namely the hippocampus and basolateral amygdala (BLA). No alterations were observed in the morphology of granule neurons in the vDG or dDG of the hippocampus (Figure 3A–H, Table S2). On the contrary, the apical dendrites of BLA pyramidal neurons of GFAP‐Tk females presented increased complexity, with increased number of nodes (Figure 3I; unpaired two‐tailed *t* test, t = 2.587, df = 6, *p* = 0.041, R^sq^ = 0.53), dendritic length (Figure 3J; unpaired two‐tailed *t* test, t = 2.698, df = 6, *p* = 0.036, R^sq^ = 0.55) and number of intersections in the Sholl analysis (Figure 3K; repeated‐measures ANOVA, for genotype: F(1,6) = 5.853, *p* = 0.051, Et^2^ = 0.49), when compared to WT females. This effect was exclusively observed in females, as no differences were observed between male WT and GFAP‐Tk rats (Figure 3I–P, Figure S3F, Table S2).

## 4 DISCUSSION

**FIGURE 4:**
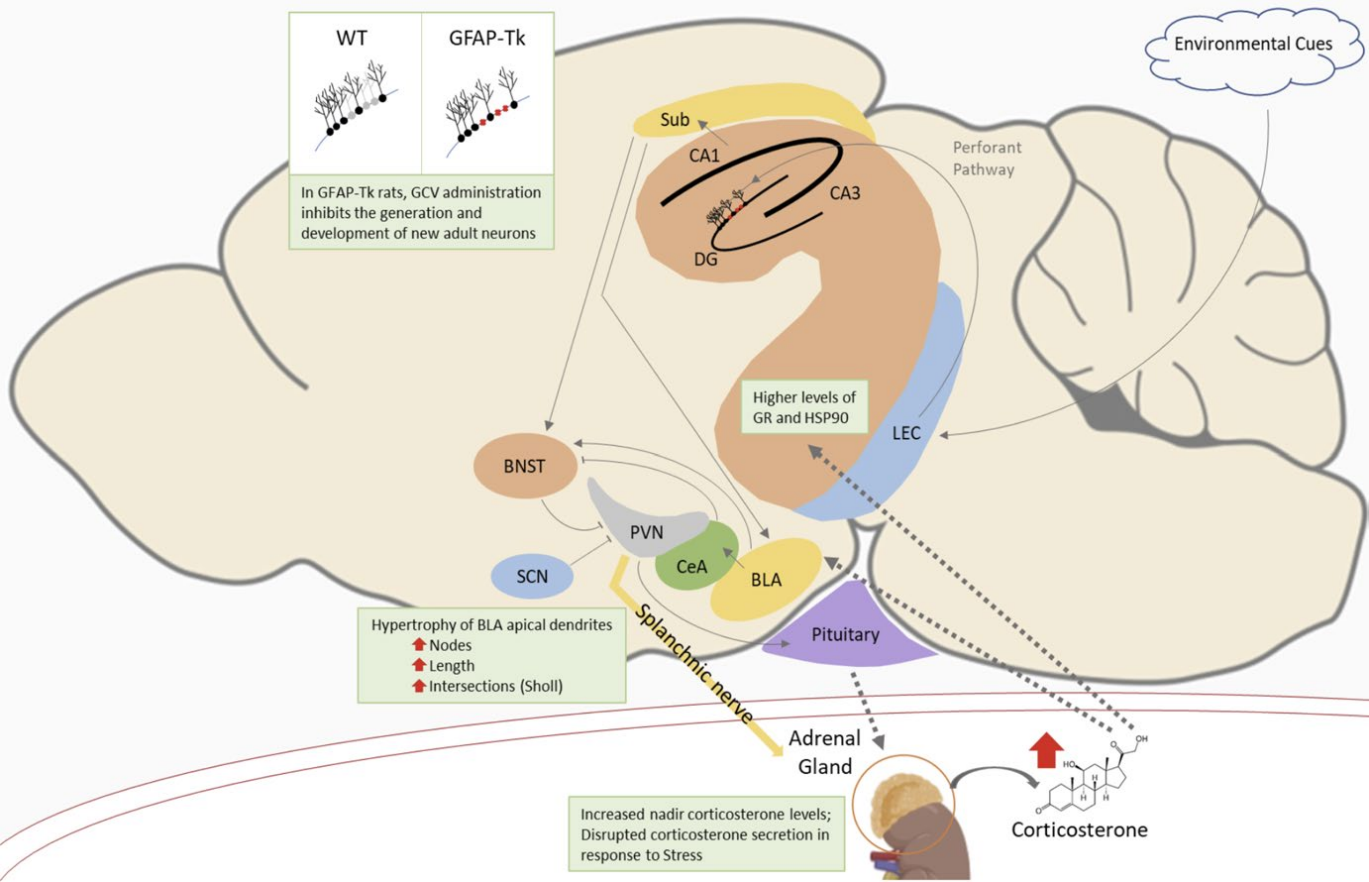
*Schematic representation of the alterations in GFAP*‐*Tk female rats after adult cytogenesis ablation*. Environmental information is sent from the lateral entorhinal cortex (LEC) through the perforant pathway to the hippocampus, where adult‐born neurons are involved in processing and integrating new memories. In their absence, as happens in adult GFAP‐Tk rats treated with ganciclovir (GCV), the hippocampal output to downstream centres is compromised. In this work, female GFAP‐Tk rats showed disrupted CORT secretion, heightened, yet delayed, stress response and BLA hypertrophy

As the environmental information arriving to the hippocampal DG is at least partially integrated and contextualized by adult‐born neurons,^40^, ^41^ the absence of such neuronal population may disrupt this information processing with implications for homeostasis. Noticeably, most studies have focused on the role of cytogenesis in males.^9^, ^18^, ^29^ To explore sex differences in this context, we used a transgenic model of cytogenesis ablation—the GFAP‐Tk rat model. Herein, we show that GFAP‐Tk female rats, but not males, have increased CORT levels at nadir, which are accompanied by a heightened, yet delayed, response to an acute stress stimulus and BLA neurons hypertrophy (Figure 4). Moreover, GFAP‐Tk male rats present a heightened acute stress response.

Hippocampal lesion studies show that hippocampal dysregulation can disrupt the HPA axis, a downstream centre under hippocampal modulatory effects.^2^ When evaluating HPA axis disruptions, it is important to identify and discriminate alterations caused by mechanisms underlying the regulation of baseline/circadian fluctuations from those evoked by a stress response. The high levels of CORT observed specifically in GFAP‐Tk females at the beginning of the day period, when they were supposed to be low, can be interpreted as a basal hyperactivity of the HPA axis. Previous studies in male rats showed that CORT circadian fluctuation is accompanied by alterations in adult cytogenesis levels in the hippocampal DG^42^ and that this fluctuation is required for the pro‐neurogenic effects of the antidepressant fluoxetine in the DG.^43^ In major depressive disorder (MDD), disrupted cortisol circadian rhythms occur in around 30–50% of the patients with one of two profiles: a flattening of diurnal cortisol rhythms, with lower values at ‘peak’ hours but higher levels throughout the day, or hypercortisolaemia, the maintenance of the circadian fluctuation though at higher levels.^1^, ^42^, ^44^, ^45^, ^46^ Also, MDD female patients with flatter cortisol slopes present a less pronounced cortisol suppression following DEX administration and greater self‐reported depression severity.^46^ It has been hypothesized that this flatter cortisol slope could be interpreted as an inhibited (or attenuated) negative feedback onto the HPA axis,^46^ whose normal functioning is partly dependent on GR and mineralocorticoid receptor (MR) levels. In the present study, the higher levels of CORT at nadir observed in female rats after cytogenesis ablation are accompanied by overexpression of GR (and its chaperone, HSP90) in the vDG. This could represent an attempt to correct those levels through a defective, yet transitory, compensatory mechanism that might be, under normal circumstances, at least partly dependent on the actions of hABN. Looking at sex differentiation in CORT and DEX profiles, it has been shown that females present higher nocturnal CORT than males^47^ and higher levels of GR mRNA in the hippocampus and PVN.^47^, ^48^, ^49^ That has also been associated with higher glucocorticoid binding capacity of GR in female rats^50^ and the capacity of DEX to suppress female nocturnal CORT levels at lower doses than males.^47^

In the bloodstream, 80%–90% of glucocorticoids bind to corticosteroid‐binding globulin (CBG), 5%–10% binds to albumin, and about 5% circulates freely.^51^, ^52^ Females present higher adrenal CBG mRNA and protein levels, given to their larger adrenal glands,^52^ and oestrogens were found to be potent inducers of the hepatic synthesis of CBG.^52^, ^53^ Additionally, the sexual dimorphism that elicits increased levels of total corticosterone in females is eliminated by CBG deficiency.^54^ It has been proposed that CBG could facilitate corticosterone export from adrenal gland in stressful situations,^52^ leading to higher stress response. Additionally, CBG does not cross the blood‐brain barrier^55^, ^56^ and corticosterone in the brain has been shown to reflect free corticosterone levels in the plasma.^57^ On the contrary, DEX was found to bind negligibly to CBG.^47^ Therefore, we could hypothesize that the higher levels of CORT in GFAP‐Tk females (in an exacerbated version of the female/male dichotomy described) do not represent higher levels of free CORT, and therefore, the rise in plasma CORT is incapable of effectively suppressing the higher levels of GR and terminating the feedback loop, revealing that hABN could be a component in the process of establishing the basal level of circulating CORT.

Interestingly, the impact of high CORT levels in females onto neurogenesis is more pronounced on the vDG,^14^ suggesting that the female vDG is more likely to respond to CORT levels. Also, chronic mild stress in rats, a protocol that produces reductions in cytogenesis levels,^23^, ^58^ has identified that females, but not males, present reduced levels of GR and MR in the hypothalamus,^59^ further supporting a sex‐differentiated receptor expression in response to stress and CORT.

Although we found higher levels of GR (and HSP90) only in the ventral DG, we cannot support the notion that ventral DG is the sole responsible for the alterations observed in this study. The dorsal hippocampus has been linked to learning and spatial memory, while the ventral hippocampus has been linked to mood and emotional processing. Recent works have further proposed a view where the hippocampus is seen as having a gradient of function and activity, with the whole structure contributing to complementary, yet distinct, functions. For example, inactivating both dorsal and ventral hippocampus resulted in a severe impairment in spatial reference memory in the water maze test.^60^ Remarkably, inactivating selectively either the dorsal or the ventral hippocampus also led to an impaired spatial memory, although with a milder phenotype. When looking specifically to the activation of hABN in the context of the Morris water maze, a classical spatial memory paradigm, Snyder et al,^61^ has shown that the dorsal DG shows higher overall c‐fos expression than the ventral DG. However, when looking at hABN, higher rates of c‐fos expression were found in the ventral DG, further supporting the notion that both poles of the hippocampus are necessary for a task classically associated with only one of them.

Regarding mood, only lesions of the ventral hippocampus (but not dorsal or intermediate hippocampus) reduced anxiety‐like behaviour and prevented the antidepressant‐like behavioural effects of fluoxetine treatment.^62^ Furthermore, specific inhibition of hABN in the ventral hippocampus caused an increase in the activity of mature granule cells in the ventral hippocampus and causes increased anxiety‐like behaviour.^63^ Future works using a similar approach to that of Lee et al^60^ would help explore whether an integrated and interdependent response is also present regarding mood.

Besides the suggested stronger relationship between vDG neurogenesis and CORT regulation, we think that the lack of hABN along the longitudinal axis of the hippocampus can potentially have a greater effect than the isolated effect of the loss of hABN specifically in the ventral pole. This hypothesis would require further studies exploring selective ablation of hABN in specific regions of the hippocampus, similar to those shown for spatial memory.^60^

Complementarily, increasing evidence shows spatial segmentation of function, neurotransmitter density and synaptic input occurs in a gradient along the longitudinal axis.^64^, ^65^ In several species and rodent strains, including in Sprague Dawley rats (the one used in this study), hABN have been reported to be in higher number in the dorsal than in the ventral hippocampus.^66^, ^67^, ^68^, ^69^, ^70^, ^71^ Moreover, ventral hABN were shown to maturate at a slower rate,^9^, ^72^ while the number of activated neural stem cells gradually decreases along the longitudinal axis (from dorsal to ventral) of the hippocampus.

Our previous assessments of this animal model show that virtually all hABN, that is along the whole longitudinal axis of the DG, are being depleted.^30^ The differences in the number of hABN along the longitudinal axis before the beginning of the treatment may indeed have an impact when assessing the effects of cytogenesis abrogation; however, based on the analyses of this study we cannot further conclude on its potential effects.

Regarding the transversal axis of the DG, increased adult neurogenesis at baseline was observed in the suprapyramidal when compared to the infrapyramidal blade.^61^, ^67^, ^68^, ^73^, ^74^, ^75^, ^76^ Differences in response to different stimuli, including stress,^77^ attributable to increased plasticity in the suprapyramidal blade,^61^ have also been identified.^64^

We also show that cytogenesis ablation, in both male and female rats, produces an exacerbated response to acute restraint stress. While, in GFAP‐Tk male rats, CORT levels were normalized after 60‐min post‐stress, a result that is further corroborated by a previous report using a male mouse model of cytogenesis ablation,^9^ female stress responsiveness appears to lag. It is unclear, though, for how long could CORT levels have remained high, as our analysis stopped at 60 min post‐stress. In general, HPA axis responses in females take longer to return to baseline levels,^78^, ^79^ further suggesting that HPA deactivation (via DEX or natural passage of time) is also intact in GFAP‐Tk females.

A study using a model of unpredictable chronic mild stress (uCMS) in the iBax mouse line (which promotes hABN proliferation) has shown a HPA axis‐disrupted response to an acute stressor in male mice.^10^ uCMS mice exposed to an acute stressor presented a lower increase in CORT levels when compared to the unstressed mice response. Interestingly, promoting neurogenic proliferation of uCMS mice to the levels of unstressed mice rescued this phenotype, further supporting that a tight regulation of hippocampal cytogenesis is needed for a proper HPA axis function. Because this study focused only on males, comparison with our study is hampered, reinforcing the importance of including both sexes in future studies.

The heightened stress responsiveness can be further explored by looking at hippocampal input and output regions. The hippocampus receives projections from the locus coeruleus, a region in the brainstem, whose sex dimorphism is described to make females more susceptible to hyperarousal symptoms,^80^, ^81^ thus making it a possible source of differentiated HPA axis activity between sexes. The hippocampus sends direct synaptic output to regions within the HPA axis, namely the BLA and the bed nucleus of the stria terminalis (BNST). BNST, which is sex‐dimorphic in adulthood, integrates inputs from various sources, including mPFC, hippocampus, BLA and central amygdala (CeA), and helps in modulating neuroendocrine and behavioural responses, including anxiety‐like behaviour.^82^, ^83^, ^84^, ^85^, ^86^ Despite not described to be sex‐dimorphic under basal conditions, BLA is hypertrophic specifically in females upon cytogenesis ablation, in this study. In males exposed to stress, BLA hypertrophy has been linked to elevated CORT levels and anxiety‐like behaviour.^87^, ^88^ Additionally, the exogenous delivery of CORT leads to BLA hypertrophy and increased anxiety‐like behaviour^89^, ^90^ and inactivating or lesioning the BLA partially prevents stress‐induced increase in CORT levels.^91^ Moreover, *in vitro* electrophysiology studies of BLA show a sex‐differentiated responsiveness to CORT.^92^ Since BLA directly stimulates BNST and inhibits it through the BLA‐CeA‐BNST pathway, disruptions at the BLA level could account for sex‐differentiated BNST outputs.

Previous reports in males^9^, ^18^, ^30^ show different outcomes in anxiety‐like behaviour. Snyder et al.^9^ showed that GFAP‐Tk male mice only exhibit disrupted anxiety‐like behaviour when previously exposed to acute restrain stress, while Groves et al.^18^ showed that GFAP‐Tk male rats exhibit reduced anxiety‐like behaviour using tests dependent on food neophobia. Previous data from our laboratory using the same animal model show that GFAP‐Tk male rats have an increased anxiety‐like behaviour,^30^ further entangling the anxiety domain exploration. Here, we used three anxiety‐focused tests and showed that GFAP‐Tk females do not have alterations in anxiety, exposing a differentiated disruption of behavioural domains that needs further exploration to disambiguate the differences derived from methodology from those derived from biology.

Here, we show that the hippocampal cytogenesis reduction initiated a cascade of events that resulted in sex‐differentiated responsiveness to acute stress and sex‐differentiated disruption of behavioural domains. Within the hippocampus, sex differences in the functional incorporation and integration of new neurons^93^ may partially explain these differential responses. Also, the active processes of masculinization and feminization in the perinatal period that specialize hippocampal functions in adulthood and promote differentiated sensitivity to environmental factors during puberty,^94^ as well as possible sex‐differentiated cell fates, as reported in the subventricular zone (SVZ) cytogenetic niche^95^ could help explain these results.

Future studies are needed to disentangle the sex specificity of our findings and to clarify the interplay between circadian corticosterone levels and ventral hippocampus neurogenesis and connectivity.

## CONFLICT OF INTEREST

The authors of the manuscript declare that they have no conflicts of interest.

## AUTHOR CONTRIBUTION

T.S.R. and P.P. maintained the GFAP‐Tk colony, performed genotyping and collected well‐being measures. T.S.R., A.M.P. and P.P. induced the model. T.S.R. and P.P. performed the CORT *in vivo* experiments. A.M.P., A.R.M.S., N.D.A., E.L.C. and P.P. performed the behavioural tests. J.S.C., J.M.M., B.A., A.R.M.S., N.D.A. and E.L.C. assisted in the *in vivo* studies and performed *in vitro* experiments, namely immunohistochemistry and CORT‐level assessment. T.S.R. and J.M.S. performed and analysed the Western blot. T.S.R., J.S.C., J.M.M and M.S. performed and analysed the neuromorphology studies. T.S.R. and P.P. reviewed the analyses and interpreted the results. A.M.P., P.P. and L.P. developed the concept and designed the study. T.S.R., P.P. and L.P. planned the experiments and wrote the manuscript. All authors discussed and revised the manuscript. J.M.B., A.J.R. and N.S. contributed to the experiments' design and critically revised the manuscript. T.S.R., P.P. and L.P. revised the final manuscript.

## Supporting information

App S1

## Data Availability

The data that support the findings of this study are available from the corresponding author upon reasonable request.
