# Supplementary material for: Suppression of adult cytogenesis in the rat brain leads to sex‐differentiated disruption of the HPA axis activity
Source: Cell Prolif. 2021 Dec 30;55(2):e13165. doi: 10.1111/cpr.13165 (PMC8828259; doi:10.1111/cpr.13165)
Supplement: Supplementary file 1 — App S1 [file CPR-55-e13165-s001.docx]

**Appendix S1**

**Suppression of adult cytogenesis in the rat brain leads to sex-differentiated disruption of the HPA axis activity**

Tiago Silveira-Rosa; António Mateus-Pinheiro; Joana Sofia Correia; Joana Margarida Silva; Joana Martins-Macedo; Bruna Araújo; Ana Rita Machado-Santos; Nuno Dinis Alves; Mariana Silva; Eduardo Loureiro-Campos; Ioannis Sotiropoulos; João Miguel Bessa; Ana João Rodrigues; Nuno Sousa; Patrícia Patrício^*^; Luísa Pinto^*^

**Supplementary Materials**

**Supplementary Materials and Methods:**

**Coat score**

Coat state was scored as follows: 1- no alterations; 2- alterations in either coat status (rough coat or coloration changes) or porphyrin presence (eyes, nose or body); 3- alteration in both coat status and porphyrin presence.

**Corticosterone levels quantification**

*Quantification of corticosterone in basal conditions*

Sampling was performed between 8 and 9 am (nadir; basal) and between 8 and 9 pm (zenith; peak), 3 days after the end of GCV treatment.

*Dexamethasone suppression test (DST) in basal conditions*

Animals were injected subcutaneously (s.c.) with dexamethasone (DEX; 30 µg.kg^-1^ in sesame oil; CAS 50-02-2; Sigma, USA) or sesame oil (vehicle) at 10 am, 3 days after the end of GCV treatment. Blood was collected on the same day at 6 pm.

*DST after acute stress*

Animals were injected s.c. with Dex (50 µg.kg^-1^) or sesame oil at 10 am, 3 days after the end of treatment. Ninety (90) min after injection, animals were subjected to a restraint stress for 60 min, by placing them in a plastic cylinder-shaped box. Blood was collected 10, 30 and 60 min after the end of the restraint stress.

**Behavioral analysis**

Novelty Suppressed Feeding (NSF) test

After a 20 h food-deprivation period, animals were placed in an illuminated enclosed arena, where a single food pellet was placed upright in the center. After reaching the pellet (either by feeding or retrieving it), animals were put in a polypropylene cage, identical to their home cage, containing a pre-weighted food pellet, and allowed to feed during 10 min.

Elevated plus-maze (EPM)

EPM was performed in a black polypropylene plus-shaped maze, with two closed arms and two opposed open arms. The animals were placed in the center of the maze, facing a pre-determined corner and allowed to explore for 5 min, while being video recorded. The percentage of time spent in the open arms was used as a measure of anxiety-like behavior.

Open field (OF)

The OF was used to assess anxiety-like behavior. Animals were placed in an illuminated enclosed arena and allowed to explore for 5 min, while a tracking software registered their position inside the arena and the number of entries in the center of the arena. The number of entries and percentage of time spent in the center of the OF arena were used as measures of anxiety-like behavior.

Forced Swimming Test (FST)

Animals were individually placed in a transparent cylinder with water (23 ºC; 50 cm of depth) during 5 min, for two consecutive days. The trial, conducted 24 h after the pretest session, was video-recorded and the immobility time was measured.

**Western blotting – subcellular fractionation**

Tissue was homogenized with Teflon homogenizer in homogenization buffer (HB; all buffers’ components are detailed in Supplementary Table 1), x6 the sample weight, and centrifuged at 1000g for 10 minutes at 4ºC to separate the cytoplasmic and nuclear fraction. The supernatant was added to a new container with x4 HB, sonicated and centrifuged for 15 min at 13300 rpm, with the resulting supernatant corresponding to cytoplasmic fraction. The nuclear fraction was obtained by resuspending the original pellet in B1 buffer and centrifuging for 10 min at 2000g at 4ºC. The resulting pellet was resuspended in 200 µL of B2 buffer, vortexed and incubated on ice for 15 min. The mixture was centrifuged at 14000g for 15 min at 4ºC and the resulting supernatant corresponded to the nuclear fraction.

Lysates were electrophoresed using sodium dodecyl sulphate–polyacrylamide (SDS–PAGE) gels (10% acrylamide) and semi-dry transferred onto nitrocellulose membranes (Trans-Blot Turbo Blotting System; Bio-Rad, USA). Membranes were blocked in 5% non-fat dry milk in Tris Buffered Saline with Tween (TBS-T) buffer and incubated with the following primary antibodies: Glucocorticoid receptor (GR; 1:500; sc-393232; Santa Cruz Biotechnology, USA; 1:500; 24050-1-AP; Proteintech, USA), heat-shock protein-90 (HSP90; 1:1000; 16F1; Enzo Life Sciences, USA), actin (1:3000; ab8224; Abcam, UK) and H3 (1:1000; ab1791; Abcam, UK). After incubation with appropriate secondary antibodies, antigens were revealed by Enhanced chemiluminescence blotting substrate (ECL; Clarity; Bio-Rad, USA). A ChemiDoc instrument and ImageLab software (Bio-Rad, USA) were used for signal quantification. All values were normalized and expressed as a percentage of control values.

**Supplementary Figure and Legends:**


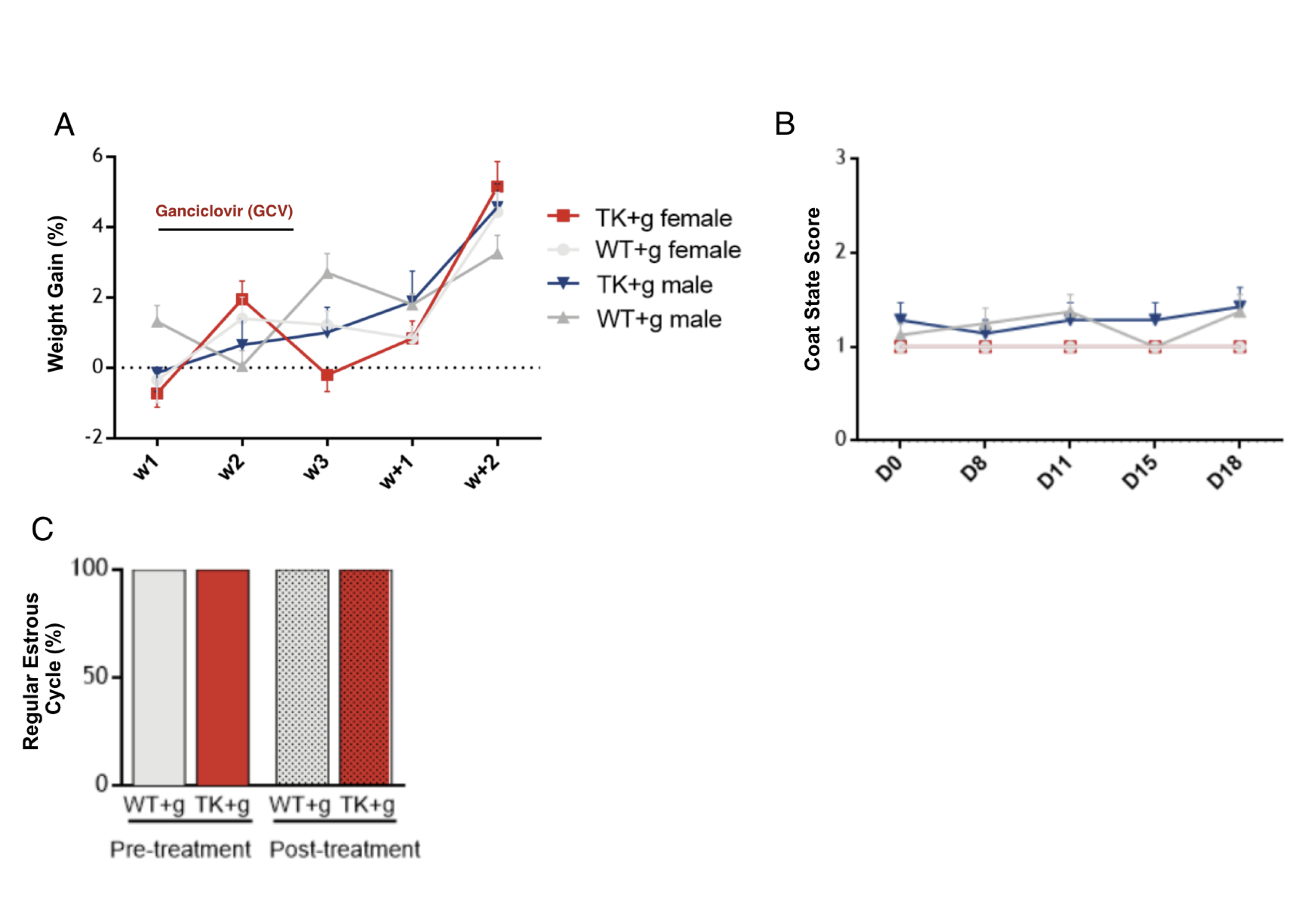


**Supplementary Figure 1.** *Well-being assessment of GFAP-Tk rat model.* a-c. General health of wild-type (WT) and GFAP-Tk rats of both sexes. a. Weight gain was unaffected. b. Coat State Score was established as follows: 1- no alteration, 2- alterations in either coat status (rough coat or coloration changes) or porphyrin presence (eyes, nose or body). 3- alteration in both coat status and porphyrin presence. Coat State Score was unaffected. c. Estrous cycle was evaluated before treatment for a week, to include at least one full estrous cycle (4-5 days). Females were considered to have regular cycles if cycle changes were in accordance with previous reports time estimates with a maximum deviation of 6h. Females estrous cycle was unaffected. Error bars denote s.e.m.


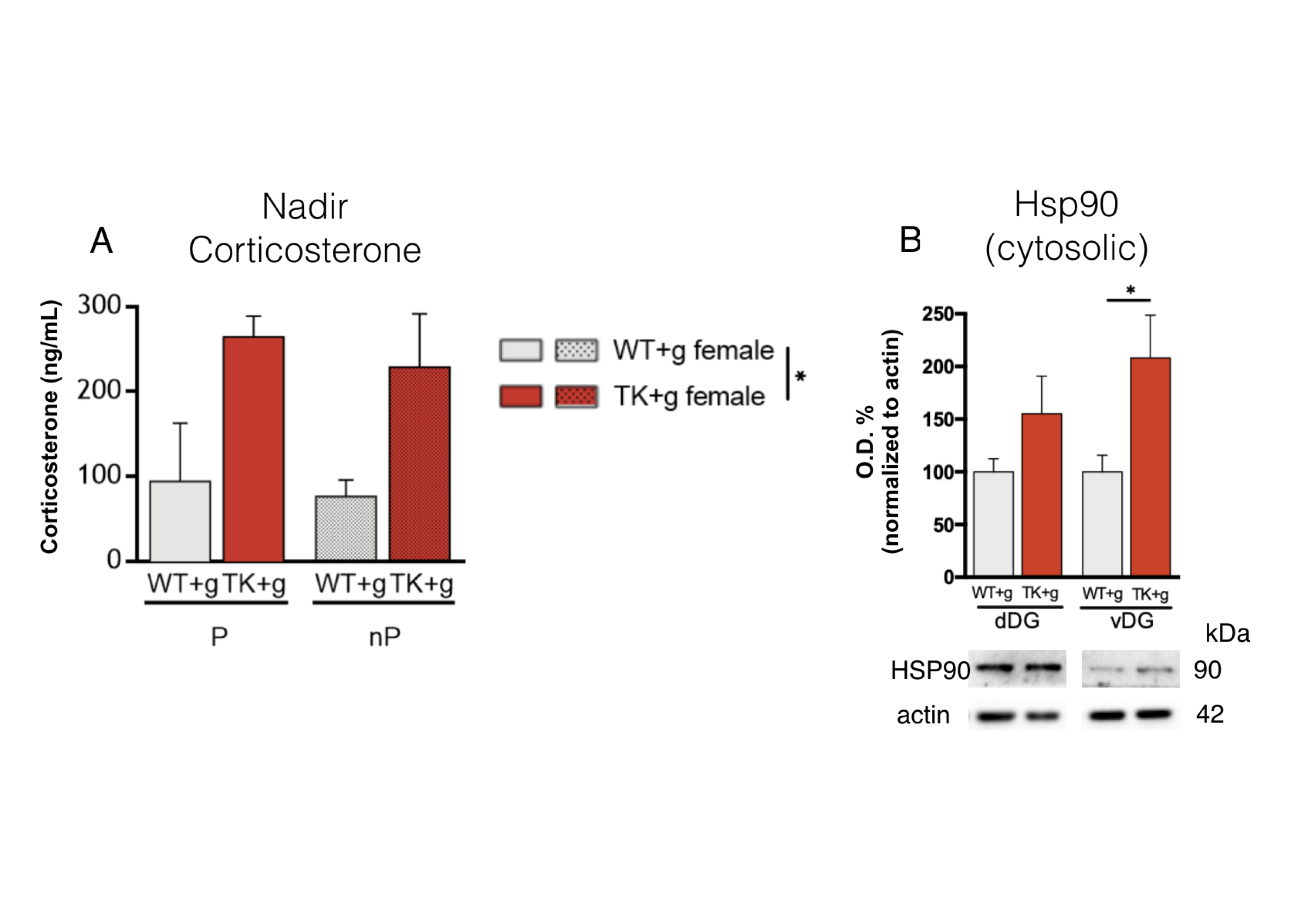


**Supplementary Figure 2.** *Estrous cycle at nadir and HSP90 expression levels.* a. Estrous cycle assessed at nadir CORT collections, divided in proestrus (p) and non-proestrus (nP). GFAP-Tk females have higher levels that WT, but estrous status did not differ with genotype. b. HSP90 expression levels. GFAP-Tk females present an increased expression of HSP90 at the ventral dentate gyrus (vDG). Error bars denote s.e.m. *P<0.05.


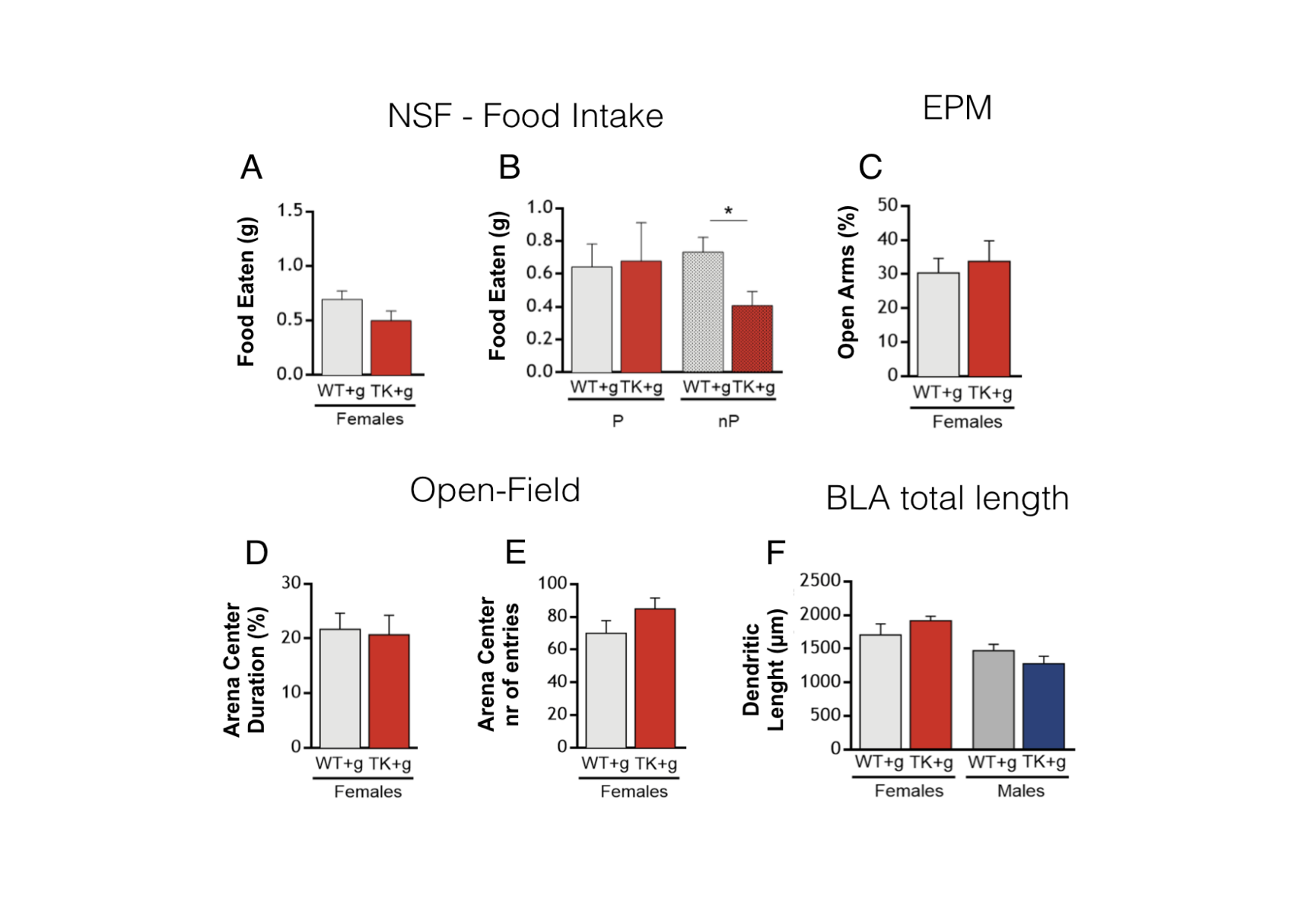


**Supplementary Figure 3.** *Behavioral outcomes and basolateral amygdala total length.* a-b. Novelty Suppressed Feeding (NSF) food intake, assessed in the 10 minutes after completing the trial. c-e. Anxiety-like behavior was measured in the Elevated Plus Maze (EPM; c) and Open-Field test (d-e). f. Basolateral amygdala (BLA) total dendritic length. No differences were present. Error bars denote s.e.m. *P<0.05.

**Supplementary Tables:**

**Supplementary Table 1 –** Western Blot Buffer Components

| HB | B1 | B2 |
| --- | --- | --- |
| Sucrose 9%;  DTT 5mM;  EDTA 2mM;  Tris pH7.4 25mM;  Complete Protease Inhibitor (Roche);  Phosphatase Inhibitor Cocktails I and II (Sigma) | Sucrose 0.32M;  EGTA 0.1M;  HEPES 1mM;  DTT 0.5mM;  Complete Protease Inhibitor (Roche);  Phosphatase Inhibitor Cocktails II and III (Sigma) | HEPES 10mM;  NaCl 400nM;  MgCl_2_ 1.5mM; EGTA 0.1mM;  DTT 0.5mM;  Glycerol 5%;  Complete Protease Inhibitor (Roche);  Phosphatase Inhibitor Cocktails II and III (Sigma) |

**Supplementary Table 2 –** Results and Statistical Data

| Experiments | Figures | Statistical Data |
| --- | --- | --- |
| CORT nadir and zenith | Fig. 1D  and  Suppl. Fig. 2A | **CORT nadir**  2-way ANOVA (sex; genotype) for sex: F(1,13)= 12.308, p= 0.004, Et2= 0.49  **CORT zenith**  2-way ANOVA (sex; genotype) for sex: sex: F(1,13)= 7.458, p= 0.017, Et2= 0.37  **CORT male WT (nadir-zenith)**  unpaired two-tailed t-test: t= 3.862, df= 7, p= 0.006, Rsq= 0.68  **CORT male GFAP-Tk (nadir-zenith)**  unpaired two-tailed t-test: t= 2.941, df= 7, p= 0.022, Rsq=0.55  **CORT female WT (nadir-zenith)**  unpaired two-tailed t-test: t= 2.464, df= 7, p= 0.043, Rsq= 0.46  **CORT female GFAP-Tk (nadir-zenith)**  unpaired two-tailed t-test: t= 0.133, df= 11, p= 0.896, Rsq< 0.01  **CORT nadir female (WT-GFAP-Tk)**  unpaired two-tailed t-test: t= 3.264, df= 9, p= 0.010, Rsq= 0.54  **CORT zenith female (WT-GFAP-Tk)**  unpaired two-tailed t-test: t= 0.476, df= 9, p= 0.645, Rsq= 0.02  **CORT estrous cycle phase (at nadir)**  2-way ANOVA (genotype; cycle phase) for genotype F (1,5) = 12.805, p = 0.016, Et2 = 0.72; for cycle phase F (1,5) = 0.031, p = 0.867, Et2 < 0.01 |
| Dexamethasone Suppression Test (DST) | Fig. 1F | **All animals**  3-way ANOVA (genotype; treatment; sex) for genotype: F(1,23)= 15.198, p= 0.001, Et2= 0.40; for treatment: F(1,23)= 27.784, p<0.001, Et2= 0.55; for sex: F(1,23)= 0.257, p= 0.617, Et2= 0.01 |
| DST and acute restrain stress | Fig. 1H | **All animals**  Repeated measures ANOVA (genotype; treatment; sex) for genotype: F(1,23)= 15.198, p= 0.001, Et2= 0.40; for treatment: F(1,23)= 27.784, p<0.001, Et2= 0.55; for sex: F(1,23)= 0.257, p= 0.617, Et2= 0.01  **Vehicle-only analysis**  Repeated measures ANOVA (genotype; sex) for genotype: F(1,10)= 10.932, p= 0.008, Et2= 0.522; for sex: F(1,10)= 0.007, p= 0.936, Et2< 0.01 |
|  |  | **Female analysis**  Repeated measures ANOVA (genotype; treatment) for genotype: F(1,15)= 9.003, p= 0.009, Et2= 0.38; for treatment: F(1,15)= 18.695, p= 0.001, Et2= 0.56; for genotype*treatment: F(1,15) = 7.628, p= 0.015, Et2=0.34  **Females at 10 min TP**  2-way ANOVA (genotype; treatment) for genotype: F(1,17)= 1.949, p= 0.181, Et2= 0.10; for treatment: F(1,17)= 12.936, p= 0.002, Et2= 0.43; for genotype*treatment: F(1,17)= 2.896, p=0.107, Et2= 0.15  **Females at 30 min TP**  2-way ANOVA (genotype; treatment) for genotype: F(1,17)= 9.636, p= 0.007, Et2= 0.39; for treatment: F(1,17)= 16.375, p= 0.001, Et2= 0.52; for genotype*treatment: F(1,17)= 8.774, p= 0.010, Et2= 0.37  **Females at 60 min TP**  2-way ANOVA (genotype; treatment) for genotype: F(1,17)= 5.838, p= 0.027, Et2= 0.26; for treatment: F(1,17)= 6.399, p= 0.022, Et2= 0.27; for genotype*treatment: F(1,17) = 2.313, p= 0.147, Et2=0.12 |
|  |  | **Male analysis**  Repeated measures ANOVA (genotype; treatment) for genotype: F(1,8)= 8.387, p= 0.020, Et2= 0.51; for treatment: F(1,8)= 13.145, p= 0.007, Et2= 0.62; for genotype*treatment: F(1,8)= 3.603, p= 0.094, Et2= 0.31  **Males at 10 min TP**  2-way ANOVA (genotype; treatment) for genotype: F(1,8)= 17.299, p= 0.003, Et2= 0.68; for treatment: F(1,8)= 41.120, p< 0.001, Et2= 0.84; for genotyope*treatment: F(1,8)= 8.813, p= 0.018, Et2= 0.52  **Males at 30 min TP**  2-way ANOVA (genotype; treatment) for genotype: F(1,8)= 6.040, P= 0.039, Et2= 0.43; for treatment: F(1,8)= 5.249, P=0.051, Et2= 0.40; for genotype*treatment: F(1,8)= 2.249, p=0.172, Et2= 0.22  **Males at 60 min TP**  2-way ANOVA (genotype; treatment) for genotype: F(1,8)= 1.517, p= 0.253, Et2= 0.16; for treatment: F(1,8)= 1.748, p= 0.223, Et2= 0.18; for genotype*treatment: F(1,8)= 0.499, p= 0.500, Et2= 0.06 |
| Western Blot | Fig. 1I  and  Suppl. Fig. 2B | **GR female dorsal DG**  unpaired two-tailed t-test: t= 1.612, df= 12, p= 0.131, Rsq= 0.18  **GR female ventral DG**  unpaired two-tailed t-test: t= 2.578, df= 12, p= 0.024, Rsq= 0.36  **GR male dorsal DG**  unpaired two-tailed t-test: t= 0.494, df= 8, p= 0.635, Rsq= 0.03  **GR male ventral DG**  unpaired two-tailed t-test: t= 1.050, df= 8, p= 0.324, Rsq= 0.12  **HSP90 female dorsal DG**  unpaired two-tailed t-test: t= 1.283, df= 12, p= 0.224, Rsq= 0.12  **HSP90 female ventral DG**  unpaired two-tailed t-test: t= 2.1.98, df= 12, p= 0.048, Rsq= 0.29 |
| Behavioral analysis | Fig. 2B-I  and  Suppl. Fig. 3A-E | **Sucrose Consumption Test**  Females - unpaired two-tailed t-test: t= 1.488, df= 24, p= 0.150, Rsq= 0.08  Estrous cycle - 2-way ANOVA (genotype; estrous) for genotype: F(1,22)= 0.848, p=0.367, Et2= 0.04; for cycle phase: F(1,22)= 0.851, p= 0.366, Et2= 0.04; for genotype*cycle phase: F(1,22)= 2.691, p= 0.115, Et2= 0.11  Males - unpaired two-tailed t-test: t= 0.417, df= 17, p= 0.682, Rsq= 0.01  **Forced Swimming Test**  Females - unpaired two-tailed t-test: t= 0.837, df= 22, p= 0.412, Rsq= 0.03  Males - unpaired two-tailed t-test: t= 0.558, df=17, p= 0.584, Rsq= 0.02  **Novelty Suppressed Feeding**  Females - unpaired two-tailed t-test: t= 0.366, df= 36, p= 0.7165, Rsq< 0.01  Estrous cycle - 2-way ANOVA (genotype; estrous) for genotype: F(1,33)= 0.007, p= 0.933, Et2< 0.01; for cycle phase: F(1,33)= 1.084, p= 0.305, Et2= 0.03; for genotype*cycle phase: F(1,33)= 10.116, p= 0.003, Et2= 0.24  Pro-estrus - unpaired two-tailed t-test: t= 2.012, df=11, p= 0.069, Rsq= 0.27  Non-proestrus - unpaired two-tailed t-test: t= 2.651 df= 22, p= 0.015, Rsq= 0.24  Males - unpaired two-tailed t-test: t=6.202, df=17, p<0.0001, Rsq=0.69  **Novelty Suppressed Feeding - food intake**  unpaired two-tailed t-test: t= 1.656, df=36, p= 0.1064, Rsq= 0.07  Estrous cycle - 2-way ANOVA (genotype; estrous) for genotype: F(1,33)= 1.317, p= 0.259, Et2= 0.04; for cycle phase: F(1,33)= 0.508, p= 0.481, Et2= 0.02; for genotype*cycle phase: F(1,33)= 2.045, p= 0.162, Et2= 0.06  Pro-estrus - unpaired two-tailed t-test: t= 0.139, df= 11, p= 0.892, Rsq< 0.01  Non-proestrus - unpaired two-tailed t-test: t= 2.609, df= 22, p= 0.016, Rsq= 0.24  **Elevated Plus Maze**  unpaired two-tailed t-test: t= 0.469, df= 12, p= 0.647, Rsq= 0.02  **Open Field Test**  Duration in arena center - unpaired two-tailed t-test: t= 0.213, df= 12, p= 0.835, Rsq< 0.01  Nr of entries in arena center - unpaired two-tailed t-test: t= 1.460, df= 12, p= 0.170, Rsq= 0.15 |
| Morphological Analysis | Fig. 3A-H | **Female dorsal DG**  Nodes - unpaired two-tailed t-test: t= 0.148, df= 6, p= 0.887, Rsq< 0.01  Length - unpaired two-tailed t-test: t= 0.386, df= 6, p= 0.713, Rsq= 0.02  Intersections - Repeated measures ANOVA (genotype; distance) for genotype: F(1,6) = 0.197, p= 0.673, Et2= 0.03  **Female ventral DG**  Nodes - unpaired two-tailed t-test: t= 0.519, df= 6, p= 0.622, Rsq= 0.04  Length - unpaired two-tailed t-test: t= 0.324, df= 6, p= 0.757, Rsq= 0.02  Intersections - Repeated measures ANOVA (genotype; distance) for genotype: F(1,6)= 0.088, p= 0.777, Et2= 0.13  **Male dorsal DG**  Nodes - unpaired two-tailed t-test: t= 0.589, df=8, p= 0.572, Rsq= 0.04  Length - unpaired two-tailed t-test: t= 1.003, df= 8, p= 0.345, Rsq= 0.11  Intersections - Repeated measures ANOVA (genotype; distance) for genotype: F(1,8)= 0.993, p= 0.348, Et2= 0.11  **Male ventral DG**  Nodes - unpaired two-tailed t-test: t= 0.300, df=7, p= 0.773, Rsq= 0.01  Length - unpaired two-tailed t-test: t= 0.205, df= 7, p= 0.843, Rsq< 0.01  Intersections - Repeated measures ANOVA (genotype; distance) for genotype: F(1,7)= 0.025, p= 0.878, Et2< 0.01 |
|  | Fig. 3I-K  and  Suppl. Fig. 3F | **Female BLA apical dendrites**  Nodes - unpaired two-tailed t-test: t= 2.587, df= 6, p= 0.041, Rsq= 0.53  Length - unpaired two-tailed t-test: t= 2.698, df= 6, p= 0.036, Rsq= 0.55  Intersections - Repeated measures ANOVA (genotype; distance) for genotype: F(1,6)= 5.853, p= 0.051, Et2= 0,49  Bonferroni multiple comparisons test: 60nm: t= 3.225, df= 144, p= 0.002; 80nm: t= 3.628, df= 144, p< 0.001; 100nm: t= 5.946, df= 144, p< 0.001; 120nm: t= 5.341, df= 144, p< 0.001  **Female BLA basal dendrites**  Nodes - unpaired two-tailed t-test: t= 0.7306, df= 6, p= 0.493, Rsq= 0.08  Length - unpaired two-tailed t-test: t= 0.454, df= 6, p= 0.666, Rsq= 0.03  Intersections - Repeated measures ANOVA (genotype; distance) for genotype: F(1,6)= 0.300, p= 0.600, Et2= 0.05  **Female BLA total dendrite length**  unpaired two-tailed t-test: t= 1.645, df= 6, p= 0.151, Rsq= 0.31 |
|  | Fig. 2M-O  and  Suppl. Fig. 3F | **Male BLA apical dendrites**  Nodes - unpaired two-tailed t-test: t= 0.346, df= 8, p= 0.738, Rsq= 0.01  Length - unpaired two-tailed t-test: t= 1.848, df= 7, p= 0.107, Rsq= 0.33  Intersections - Repeated measures ANOVA (genotype; distance) for genotype: F(1,7)= 1.091, p= 0.331, Et2= 0.13  **Male BLA basal dendrites**  Nodes - unpaired two-tailed t-test: t= 0.965, df= 8, p= 0.363, Rsq= 0.10  Length - unpaired two-tailed t-test: t= 1.212, df=8, p= 0.260, Rsq= 0.16  Intersections - Repeated measures ANOVA (genotype; distance) for genotype: F(1,8)= 2.064, p= 0.189, Rsq= 0.21  **Male BLA total dendrite length**  unpaired two-tailed t-test: t= 1.317, df= 7, p= 0.229, Rsq= 0.20 |
| Well-Being Assessment | Suppl. Fig. 1A-B | **Female weight variation**  Repeated measures ANOVA (genotype; time) for genotype: F(1,39)= 0.316, p= 0.578, Et2< 0.01  **Male weight variation**  Repeated measures ANOVA (genotype; time) for genotype: F(1,23) = 0.491, p= 0.490, Et2= 0.02  **Female Coat Score**  No variation was observed (all scores = 1)  **Male Coat Score**  Repeated measures ANOVA (genotype; time) for genotype: F(1,13)= 0.156, p= 0.6989, Et2= 0.02 |
